# Small bowel bleeding: a comprehensive review

**DOI:** 10.1093/gastro/gou025

**Published:** 2014-05-29

**Authors:** Deepak Gunjan, Vishal Sharma, Surinder S Rana, Deepak K Bhasin

**Affiliations:** Department of Gastroenterology, Postgraduate Institute of Medical Education and Research (PGIMER), Chandigarh, India

**Keywords:** small intestine bleed, aetiology, diagnostic approach, management

## Abstract

The small intestine is an uncommon site of gastro-intestinal (GI) bleeding; however it is the commonest cause of obscure GI bleeding. It may require multiple blood transfusions, diagnostic procedures and repeated hospitalizations. Angiodysplasia is the commonest cause of obscure GI bleeding, particularly in the elderly. Inflammatory lesions and tumours are the usual causes of small intestinal bleeding in younger patients. Capsule endoscopy and deep enteroscopy have improved our ability to investigate small bowel bleeds. Deep enteroscopy has also an added advantage of therapeutic potential. Computed tomography is helpful in identifying extra-intestinal lesions. In cases of difficult diagnosis, surgery and intra-operative enteroscopy can help with diagnosis and management. The treatment is dependent upon the aetiology of the bleed. An overt bleed requires aggressive resuscitation and immediate localisation of the lesion for institution of appropriate therapy. Small bowel bleeding can be managed by conservative, radiological, pharmacological, endoscopic and surgical methods, depending upon indications, expertise and availability. Some patients, especially those with multiple vascular lesions, can re-bleed even after appropriate treatment and pose difficult challenge to the treating physician.

## INTRODUCTION

Gastro-intestinal (GI) bleeding is a common and perplexing problem encountered by gastroenterologists. The small intestine is the least common site of GI bleeding but is the commonest cause of obscure GI bleed. It is estimated that upper gastro-intestinal bleeding (UGIB) (from the oesophagus to duodenum), lower gastro-intestinal bleeding (LGIB) (from the colon and anorectum) and obscure bleeding account, respectively, for 50%, 40% and 10% of total GI bleeding [1]. The small bowel is called ‘the dark continent of the GI tract’ because of its inaccessibility to endoscopists, due to its intra-peritoneal location, excess mobility and long length. Approximately 5% of GI bleeding occurs from the small bowel, defined as the region between the ligament of Treitz and the ileocecal valve [2]. Traditionally, various reports have included small bowel bleeding in LGIB (distal to the ligament of Treitz) or as a cause of obscure gastro-intestinal bleeding (OGIB). Recent advances have led to reclassification of GI bleeding into three categories: upper-, mid- and lower GI bleeding. If the source of GI bleeding is between the ampulla of Vater and the terminal ileum, it is designated as mid-GI bleeding [3, 4]. Because of an inability to visualize the small bowel properly, patients with a small bowel GI bleed usually end up undergoing multiple diagnostic investigations, requiring multiple hospitalisations and transfusions; therefore, it is necessary to identify the cause and site of haemorrhage accurately, so as to institute appropriate, effective therapy.

In last decade, the availability of advanced diagnostic innovations like capsule endoscopy (CE), double-balloon enteroscopy (DBE) and computed tomography enterography has led to better understanding of the aetiological profile of small bowel bleeding and there is a paradigm shift in the management of small bowel bleeding, with the majority of cases now being treated non-surgically. In this review, we will discuss the aetiology, current diagnostic approach and the therapeutic options available for managing patients with small bowel bleed.

## AETIOLOGY

A variety of lesions may result in small bowel bleeding, with the aetiology of bleeding being different in various age groups (Table 1). The commonest lesions responsible for small bowel GI bleeding are vascular, with other causes being tumours, inflammatory lesions, and medications, as well as some rare causes like haemobilia, *haemosuccus pancreaticus* and aorto-enteric fistula. Vascular lesions and small bowel lesions induced by non-steroidal anti-inflammatory drugs (NSAID) are the common causes of small bowel GI bleeding in the elderly, whereas tumours, Meckel’s diverticulum, Dieulafoy’s lesion and Crohn’s disease are the common causes in patients under 40 years of age [5, 6]. Zhang *et al.* studied 385 OGIB patients and found that, in elderly patients (>65 years), vascular anomalies (54.35%), small intestinal ulcer (13.04%), small intestinal tumours (11.96%) were the common cause of small intestinal bleeding; in middle age (41–64 years) vascular anomalies (34.82%), small intestinal tumours (31.25%), non-specific enteritis (9.82%) were the major causes and in young adults (<40 years), the leading causes were Crohn’s disease (34.55%), small intestinal tumours (23.64%) and non-specific enteritis (10.91%) [6].

**Table 1.** Etiology of obscure gastro-intestinal bleeding according to age^5^^,^^6^

Elderly	Middle-Aged	Young Adult
(>65 years)	(41-65 years)	(17-40 years)
Vascular anomalies Small intestinal ulcer NSAID enteropathy Small intestinal tumours Non-specific enteritis Celiac disease	Vascular anomalies Small intestinal tumours Non-specific enteritis Small intestinal ulcer	Crohn’s disease Small intestinal tumours Meckel’s diverticulum Non-specific enteritis Dieulafoy’s lesion Vascular anomalies Celiac disease

The various small bowel vascular anomalies described include angiodysplasia, telangiectasia, phlebectasia, arteriovenous malformation (AVM), Dieulafoy’s lesion and varices.

**Figure 1.**
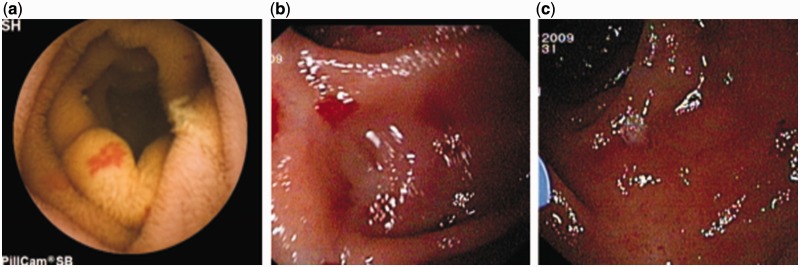
Patient of small bowel bleeding due to angiodysplasia in jejunum a: Capsule endoscopy: angiodysplasia in jejunum; b: Enteroscopy: angiodysplasia in jejunum; c: Argon plasma coagulation (APC) of angiodysplasia.

**Angiodysplasia** (angioectasia or vascular ectasia) is abnormally dilated, tortuous, thin-walled vessels, involving small capillaries, veins and arteries (Figures 1a, 1b, 1c) [7, 8]. They are visualized within the mucosal and submucosal layers of the gut, are lined by endothelium with little or no smooth muscle, and lack inflammatory or fibrotic changes as well as fibrosis [8, 9]. They are the most common cause of small bowel bleeds. In a systematic review by Liao *et al.* that included 227 studies and 22 840 small bowel capsule endoscopies, OGIB, at 66%, was the most common indication and angiodysplasia was the most common underlying lesion (50%) [10]. Meyer *et al.* reviewed 218 cases of arterio-venous malformations (AVM) and found that the cecum or right colon was the most common location (78%), whereas the jejunum (10.5%), ileum (8.5%) and duodenum (2.3%) are other sites for AVM [11].

Angiodysplasia is associated with various clinical conditions and syndromes. Bleeding from angiodysplasia in patients with aortic stenosis (AS)—termed Heyde's syndrome—is a well-known clinical syndrome [12–16]. It has been shown that high stress in aortic stenosis causes shear-dependent cleavage of high molecular weight multimers of von Willebrand’s factor (vWF), leading to acquired vWF deficiency [17]: vWF is essential for the adhesion and aggregation of platelets to the sub-endothelium of damaged blood vessels. Aortic valve replacement had ameliorated the acquired vWF abnormality, suggesting an association between them [17]. An American Gastroenterology Association technical review in 2007 concluded that, even if there is an association between aortic stenosis and angiodysplasia, it is weak and often exaggerated [5]. It is proposed that patients with AS have previously non-recognised latent intestinal angiodysplasia that bleeding as a result of this acquired haematological defect.

Chronic renal failure (CRF) is another condition that is associated with increased frequency of GI angiodysplasia. Karagiannis *et al.* studied 17 CRF patients and 51 patients with normal renal function who had presented with OGIB; 47% of patients with CRF had small bowel angiodysplasia as compared with 17.6% of those with normal renal functions [18]. Another study of patients who were on haemodialysis made similar observations and these lesions were found to be more common in the ileum [19]. Uremic platelet dysfunction is one of the supposed mechanisms for increased risk of bleeding in patients with CRF [20]. In a recent study, hypertension, ischaemic heart disease, arrhythmias, valvular heart disease, congestive heart failure, chronic respiratory conditions, previous venous thrombo-embolism, and use of anti-coagulants have been found to be risk factors associated with small bowel angiodysplasia [21].

**Telangiectasias** usually have associated cutaneous and mucous membrane involvement, in contrast to angiodysplasias where only the GI tract mucosa is involved. Telangiectasias lack capillaries and consist of direct connections between arteries and veins and have excessive layers of smooth muscle without elastic fibres [22]. Hereditary haemorrhagic telangiectasia (HHT) is a condition commonly associated with small intestinal telangiectasia; it usually presents with epistaxis in younger age and GI bleeding is a delayed manifestation that usually does not occur until the fifth or sixth decade of life [23]. Telangiectasias occur throughout the GI tract, but are more common in the stomach and duodenum and these patients usually present with iron deficiency anaemia (IDA), with recurrent GI bleeding occuring in 15–20% [24, 25]. Turner’s syndrome and scleroderma are other clinical conditions that are associated with GI telangiectasia [26, 27].

**Dieulafoy’s lesion** is a rare cause of GI bleeding that can sometimes be massive and life-threatening [28]. It is most commonly located in the stomach and the small bowel is an uncommon site [28]. In a study by an Austrian group in 284 patients with mid GI bleed, Dieulafoy’s lesion was found in 10 patients (3.5%) (in the proximal jejunum in nine patients and the ileum in one patient) [29]. Nortan *et al.* studied 4804 episodes of acute GI bleeding, in which they identified 90 Dieulafoy’s lesions in 89 patients, but only 2% of these lesions were located in the jejunum [30]. In another review of 249 cases of Dieulafoy’s lesion, only 26 cases (were identified in the small bowel (beyond the ligament of Trietz) [31].

**Small bowel varices** are large, portosystemic venous collaterals occurring in the small intestine; they are most commonly associated with portal hypertension or abdominal surgery and are uncommon causes of GI bleeding [32]. In a review of 169 patients with bleeding ectopic varices, 17% of patients had varices in the duodenum, 17% in the jejunum or ileum and 26% of patients bled from peristomal varices [32]. In a survey by the Japan Society of Portal Hypertension, which included 173 patients of ectopic varices, duodenal, jejunal, ileal, and peristomal varices were present in 32.9%, 4%, 1.2% and 5.8% of patients, respectively [33]. A study of 37 patients with cirrhosis and portal hypertension who underwent CE reported that small bowel varices were seen in 8.1% of patients [34]. In a report from our centre, of 41 patients with portal hypertension who underwent ileocolonoscopy, ileal varices were observed in 21%; also one-third of these patients had ileal mucosal changes suggestive of portal hypertensive ileopathy [35].

**Small bowel tumours** are uncommon causes of GI bleeding, with primary small bowel tumours comprising about 5% of all primary GI tract neoplasm [36]. Small bowel tumours have been reported to be the second most common cause of small bowel bleeding, accounting for 5–10% of cases [37]. In a series of 49 patients, Ciresi *et al.* reported that benign tumours more commonly presented with acute GI haemorrhage (29% vs 6%), and were more often asymptomatic (47% vs 6%) as compared with malignant small bowel tumours [38]. Adenocarcinoma is the most common primary malignancy of the small bowel, accounting for 35–50% of small bowel tumours, whereas carcinoid tumours account for 20–40%, lymphomas 14%, and sarcomas 11–13% [39, 40]. Adenocarcinomas are more common in the duodenum and proximal jejunum, whereas lymphomas and carcinoid tumours are most frequently located in the distal small bowel [40–43]; the sarcomas are evenly distributed throughout the small bowel. Data from the Connecticut Tumor Registry reported that the most common location of small bowel tumours was the ileum (29.7%), followed by the duodenum (25.4%) and the jejunum (15.3%) [44].

**Gastro-intestinal stromal tumours (GIST)** probably originate from the interstitial cells of Cajal, with the stomach being the most common location (50–60%) followed by the small intestine (30–35%) [45]. GI bleeding is usually due to the compression, ischaemia or infiltration of the overlying mucosa by these submucosal tumours. In a series of 47 patients with GIST, acute abdomen and small bowel bleeding were the common presenting symptoms [46]. Another study by Vij *et al.* reported GI bleeding to be the most common clinical presentation of GIST (69.6%) and the jejunum (17.4%) was the most common site, followed by the ileum (6.6%) and duodenum (3.3%) [47]. Other neoplasms that can be seen in the small bowel are leiomyoma, enteropathy-associated T-cell lymphoma (EATL), Kaposi sarcoma, polyposis syndromes and metastasis to the small bowel [48–52].

**Figure 2.**
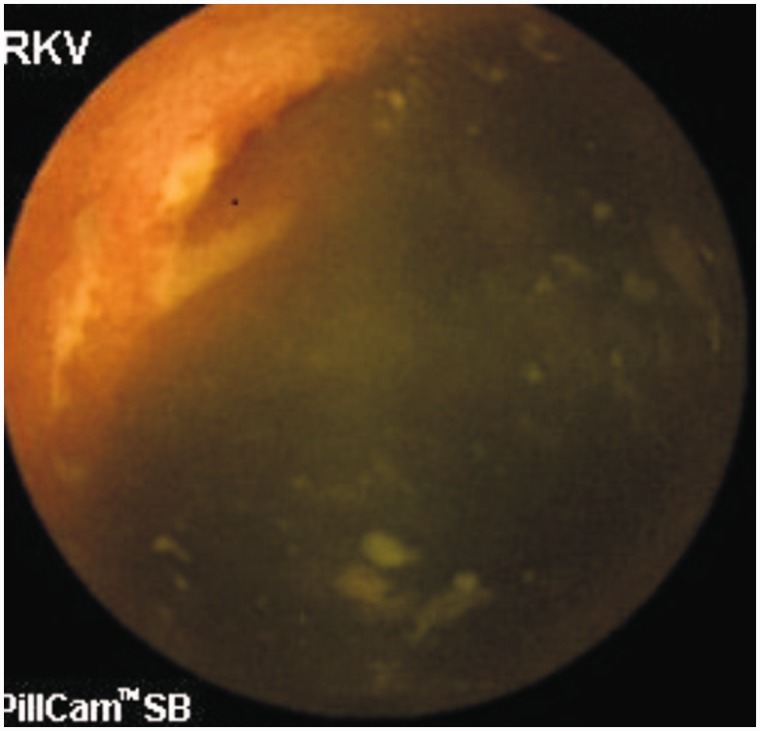
Capsule endoscopy: Small ulcers in Crohn’s disease.

**Figure 3.**
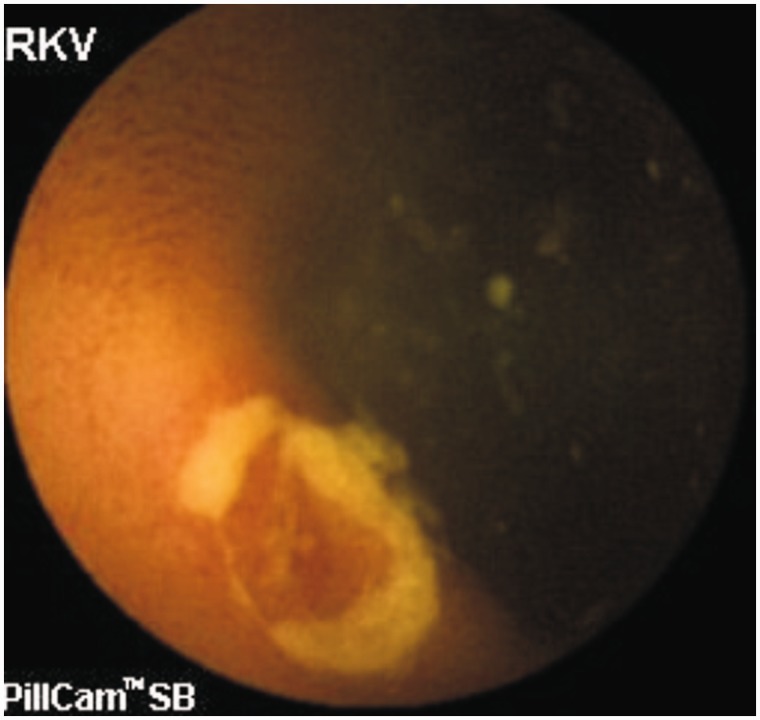
Capsule endoscopy: Large ulcer in Crohn’s disease.

**Figure 4.**
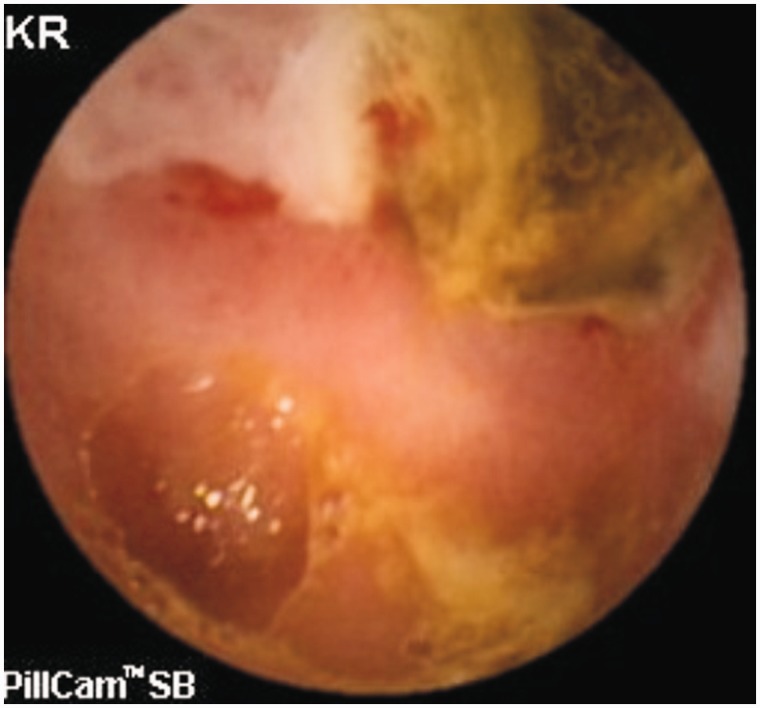
Capsule endoscopy: Ulcer with narrowing in intestinal tuberculosis.

**Figure 5.**
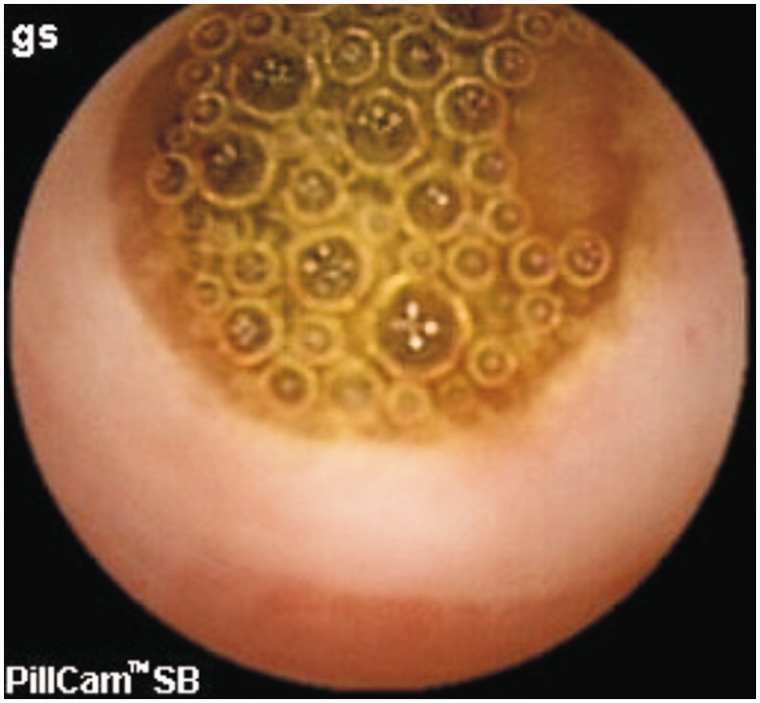
Capsule endoscopy: Ulcer with diaphragm in NSAID abuse.

**Small bowel ulcers** are another important cause of GI bleeding. Although most studies report angiodysplasia as the commonest cause of OGIB, one study involving 385 OGIB patients from India reported small bowel ulcers or erosions (156 patients) as the most common cause of OGIB [53]. The authors were not able to characterize all the ulcers, but Crohn’s disease (Figures 2 and 3), intestinal tuberculosis (Figure 4) and NSAID-induced small bowel ulcers (Figure 5) were responsible for OGIB in 42, 12, and 12 patients, respectively [53]. The prevalence of small bowel ulcers increases with age, with reported frequency of 13.04% in patients over 65, as compared with 7.27% in patients under 40 years [6]. Apart from Crohn’s disease, tuberculosis and NSAID enteropathy, other causes of small bowel ulcers could be tumours, medications, non-specific ulcers, idiopathic chronic ulcerative enteritis and celiac disease [54–56].

**Figure 6.**
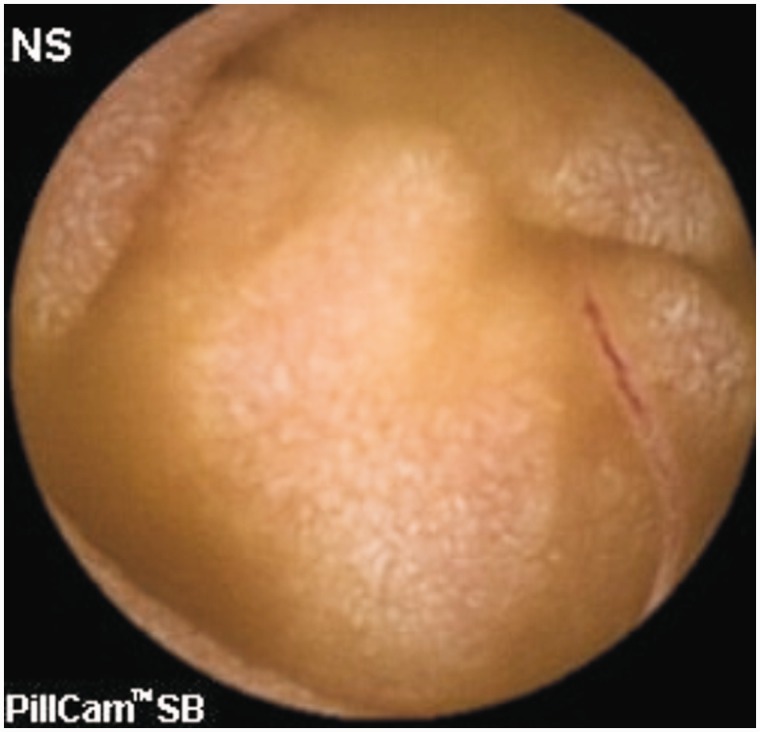
Capsule endoscopy: Hookworm.

Various infections of the small bowel, including tuberculosis, enteric fever and parasitic infections like hookworm (Figure 6) can also cause small bowel bleeding. In a study involving 40 OGIB patients, small bowel tuberculosis was responsible for bleeding in 10% [57]. GI bleeding can also occur in enteric fever and one study on an outbreak of 3010 cases of enteric fever reported melena in 38% of the patients, with the ileocecal area being the most common site of involvement (72%), and the ileum was involved in only 3% of patients [58].

**Meckel’s diverticulum** is a common congenital abnormality of the small bowel—a result of incomplete closure of the vitelline duct—and affects 2–3% of the population [59]. The bleeding usually results from the ulceration of the ectopic gastric mucosa within the diverticulum. In the Mayo Clinic, experience of 1476 patients with Meckel’s diverticulum, seen from 1950 to 2002, only 16% of patients were symptomatic, with GI bleeding being the most common presentation in adults (38%) [60]; its diagnosis is difficult and the technetium-99m (^99m^Tc) pertechnetate scan (Meckel’s scan) has a sensitivity and specificity of 85% and 95%, respectively [60]; however, in adults the sensitivity of Meckel’s scan falls to 63% because of the presence of lesser gastric mucosa in the diverticulum, as compared with children (63% vs 78%) [60–62]. False-negative results can also occur because of inadequate gastric mucosal cells, inflammatory changes causing oedema or necrosis, presence of outlet obstruction of the diverticulum or low haemoglobin levels [63, 64]. In false-negative cases, mesenteric arteriography or DBE can help in achieving a correct diagnosis. Zheng and co-workers reported 28 children with OGIB who had negative Meckel’s scan and, in 10 patients, Meckel’s diverticulum was diagnosed by DBE [63]; CE is another diagnostic modality, but there is risk of capsule retention [65, 66].

**Jejuno-ileal diverticula** are uncommon causes of small bowel bleeding, with reported frequency of 1.1–2.3% [67, 68]; they usually occur at the mesenteric border, are usually multiple and more common in the jejunum. The majority of these diverticula are asymptomatic, with GI bleeding occurring in only 3.4–8.1% of patients with small bowel diverticula, but whenever the bleeding occurs it is usually massive and recurrent [69–71].

**Aortoenteric fistula** is a rare, life-threatening condition and almost always seen secondary to reconstructive aortic aneurysmal surgery. It typically involves third portion of the duodenum and presents with herald bleeding, followed by massive life-threatening bleeding [72, 73].

**Haemobilia** is a rare cause of OGIB and is due to abnormal communication between the vessels and the biliary system. It is difficult to diagnose, however it should be suspected in any patient with a prior history of blunt trauma of the abdomen or medical procedures [74]. It usually presents as melena (90%), haematemesis (60%), biliary colic (70%) or jaundice (60%) [75]. The bleeding can occur periodically, and it can also manifest itself as massive GI haemorrhage. In recent studies the most common cause of haemobilia is iatrogenic trauma (65%), whereas earlier studies had reported accidental trauma (38.6%) as the most common cause of haemobilia [76]. Side-viewing endoscopy can directly visualize the clot extrusion or blood oozing from the papilla of Vater. Cross-sectional imaging can also help in diagnosis by showing presence of blood in the gall bladder and the biliary tree, and can also recognise various causes of haemobilia. Endoscopic retrograde cholangiopancreatography (ERCP) is rarely helpful in discerning the source of haemobilia but may be helpful in clearing the blood clots and relieving the biliary obstruction. Selective visceral arteriography of the celiac axis or superior mesenteric artery is the most definitive investigation and also has therapeutic potential [76].

**Haemosuccus pancreaticus** is another unusual cause of GI bleeding from the peripancretic blood vessel into the pancreatic duct. It usually occurs in the setting of chronic or acute pancreatitis [77]. Other causes are various tumours, vascular lesions, congenital anomalies, iatrogenic or trauma [77]. Patients typically present with intermittent epigastric pain in the abdomen, GI bleeding (melena, haematemesis, haematochezia) and hyperamylasemia. The bleeding may be intermittent or sometimes massive. The diagnostic strategy is similar to haemobilia. Selective arteriography of the celiac trunk and superior mesenteric artery is the most sensitive diagnostic tool, with sensitivity of up to 96% [78].

There are various other causes of small bowel bleeding, such as radiation enteritis, mesenteric ischaemia, and endometriosis [79]. Additionally, in tropical countries, intestinal infestation by worms can be an important cause. One study from India, in which 21 cases of obscure GI bleeding were evaluated by push enteroscopy, found worms as the cause in 28.5% of patients [80]; therefore a proper stool examination should be done in patients from tropical countries before undertaking further invasive and expensive investigations.

## MANAGEMENT OF SMALL BOWEL BLEED

### Assessment

The initial assessment of the patient with small bowel bleeding must include a good clinical history and physical examination. A small bowel bleed may present as occult- (IDA) or overt (melena or haematochezia) bleeding. It may be persistent or recurrent and can be massive, leading to shock. The history should include details of medications (NSAID, aspirin, and anticoagulants), radiation therapy, any coagulation disorder or cirrhosis, trauma, prior surgery, and recent endoscopic intervention. Family history of bleeding, recurrent epistaxis and cutaneous telangiectasia may suggest HHT. Pigmented lip lesions and family history of polyposis may point towards a diagnosis of Peutz Jegher’s syndrome and the presence of spider angiomata and caput medusa will suggest portal hypertension. History of chronic pancreatitis should be sought, which may be a clue to *haemosuccus pancreaticus*. The classical triad (the Sandblom triad) of haematemesis, upper abdominal pain and jaundice may points toward haemobilia. Painless bleeding may suggest vascular lesions, whereas painful bleeding may be due to small bowel tumours or NSAID-related GI injury. As stated earlier, history of passage of worms must be carefully looked for, especially in tropical regions.

### Diagnosis

Small bowel bleeding localization used to be a tedious and a tough job for gastroenterologists but, due to recent advances in imaging techniques, there is a paradigm shift in evaluation of patients with small bowel bleeding. Newer techniques such as capsule endoscopy, double-balloon enteroscopy, single-balloon enteroscopy (SBE), spiral enteroscopy (SE) or computed enterography play a key role in the diagnosis of small bowel bleeding.

**Small bowel radiography** used to be the main diagnostic modality for evaluating patients with small bowel bleeding but, with the advent of deep enteroscopy and newer cross-sectional imaging modalities, its use is declining. The diagnostic yield of small bowel radiography has been reported to be 5–10% in patients with suspected small bowel bleeding [81, 82]. In a meta-analysis, the yields of small bowel barium radiography were 8% for any findings and 6% for clinically significant findings; in contrast, the yields of CE were 67% and 42%, respectively [83]. Small bowel radiography is unlikely to be helpful in diagnosing vascular lesions, which are the most common cause of small bowel bleed, but may help in localizing mucosal lesions in inflammatory bowel disease, tuberculosis, ulcers or small bowel tumours.

The development of **CT enterography (CTE)** has led to improved imaging of the small bowel and its surrounding structures but, for good visualisation and better delineation of mural details, it is essential to have adequate bowel luminal distension with neutral oral contrast. It can also help in localisation of active bleeding as the presence of active GI bleeding would be seen as a focal area of hyperdense attenuation in the bowel lumen on plain scan or as focal area of contrast enhancement or extravasation into the lumen on a contrast-enhanced study [84]. Lee and co-workers evaluated the diagnostic performance of CTE in 65 patients of OGIB [85]. The sensitivity, specificity, positive predictive value, and negative predictive value of CT enterography in diagnosing the underlying cause was 55.2%, 100%, 100%, and 71.1%, respectively, and history of massive bleeding was associated with a higher diagnostic yield of CTE.

When CTE is compared with CE, the results are contradictory. One study reported a low detection rate of CTE in comparison to diagnostic yield of CE (30.08% vs 57.72%) [86]. However another study reported a higher detection rate for CTE when compared with CE (88% vs 38%, respectively) [87]. The limitation of CTE is that it cannot diagnose flat lesions such as ulcers, superficial erosions, and vascular lesions (angiodysplasias or AVM) [88]; but CTE detects small bowel tumours better, which can sometimes be missed by CE, especially those tumours with a predominantly exophytic component [89].

**Multi-detector CT angiography** does not require bowel loop distension and is performed using intravenous contrast agent. It identifies the site of active bleeding as a focal area of hyperattenuation or contrast extravasation in the bowel lumen and has a higher sensitivity in detecting active GI bleeding than for OGIB [90]. Helical CT angiography has been shown to be more sensitive than mesenteric angiography in detecting active haemorrhage, with bleeding rates as low as 0.3 mL/min being detected in animal models. This is better than the detection threshold of 0.5 mL/min of mesenteric angiography and is close the detection threshold of 0.2 mL/min of RBC scintigraphy [91]. In a meta-analysis, pooled sensitivity and specificity of CT angiography in acute GI bleeding were reported to be 89% and 85% respectively [92]. However, inability to perform therapeutic procedures is a major limitation of CT angiography.

**Catheter angiography or digital subtraction angiography (DSA)** is performed by intra-arterial injection of contrast following selective and super-selective cannulation of visceral arteries. A higher diagnostic yield of 61–72% has been reported in patients with active bleeding, in contrast to a low diagnostic yield of <20% in patients with inactive bleeding [93]. The advantage of this technique is the ability to perform embolization of bleeding vessels by various tools, such as absorbable gelatine pledgets, polyvinyl alcohol, microspheres, cyanoacrylates or microcoils, used alone or in combination. The success rate approaches 100% if active bleeding site is identified, with a complication rate of less than 5% [94]. To increase the diagnostic yield, provocative angiography has also been described by administration of heparin, thrombolytics and vasodilators that provoke bleeding and thus increase the yield of angiography [95]; however, with the availability of better imaging tecniques, such a risky approach is usually not recommended.

**Radionuclide imaging** is performed with ^99m^Tc-labelled red blood cells and it detects active bleeding. Any extravasations of radionuclide from the vascular space can be recognised as an area of concentration and slower clearing of the activity against the background [94]. Bleeding rates as low as 0.1 mL/min can be identified and the accuracy of scintigraphy in localizing bleeding varies from 40 to 100%. However, inaccurate localization of the bleeding site is observed in 25% of patients and this is one of the most important limitations of scintigraphy [96, 97]. The advantages of scintigraphy include its easiness to perform, require no patient preparation, is well tolerated and has high sensitivity in low bleeding rates. Its role in Meckel’s diverticulum is already highlighted in previous section.

**Push enteroscopy (PE)** can also help in diagnosing causes of small bowel bleeding especially if located in the proximal small bowel. Although, non-therapeutic Sonde enteroscopy can visualize the whole intestine, but it takes many hours to complete, and is now not used by most of the centres. PE allows both diagnostic and therapeutic applications, and with its help distal duodenum and proximal jejunum of variable length can be visualized. In a study of 21 patients of OGIB PE detected lesions causing GI bleeding in 42.8% of patients [80]. Another study of PE in 63 patients of OGIB found its diagnostic yield to be 41% in recurrent obscure-overt bleeding, 33% in persistent obscure-overt bleeding and 26% in obscure-occult bleeding [98].

**Capsule endoscopy (CE)** enables the complete small bowel visualization non-invasively (Figure 1-6) but currently this investigation has no therapeutic potential. In a study of 685 patients of acute overt GI bleeding (melena, haematochezia, or haematemesis) no aetiology could be found out in 37 patients after upper and lower endoscopic evaluation and these patients were subjected to capsule endoscopic examination. The diagnostic yield of CE was found out to be 91.9% and it changed the management plan in 21 patients [99]. Carey *et al.* evaluated 260 patients with OGIB and found the diagnostic yield of CE to be 53% and this was higher in patients with obscure-overt (60%) GI bleeding as compared with patients with obscure-occult GI bleeding (46%) [100]. There were significant reductions in hospitalizations, additional tests/procedures, and units of blood transfused after medical interventions in the group of patients who underwent CE [95]. In one study, CE changed the treatment strategy of physicians in 31% of patients; whereas in another study, it had a major impact in patient management in one third of the patients [101,102]. In a systemic review, the pooled diagnostic yield of CE in studies that focused solely on patients with iron deficiency anemia (IDA) was 66.6% and it detected more vascular (31% vs 22.6%), inflammatory (17.8% vs 11.3%), and mass/tumour (7.95% vs 2.25%) lesions in patients who had IDA as compared with those who did not have IDA [103].

The diagnostic yield of CE has been reported to be higher than that of barium radiography (42% vs 6% respectively, p < 0.00001), CT angiography (CTA) (72 % vs 24%, p = 0.005) and standard mesenteric angiography (72% vs 56%, p > 0.05) [83]. CE also detects lesions in patients with negative findings on CTA (63%) and standard mesenteric angiography (55%) [104]. In a prospective randomized study of patients with acute overt OGIB (n = 60) comparing immediate CE with angiography, the diagnostic yield of CE was found to be significantly higher (53.3% vs 20%, p = 0.016). However, immediate CE did not have any impact on long–term outcomes including further transfusions, hospitalization for rebleeding, and mortality [105]. Milano *et al.* prospectively compared CE with CT enteroclysis in 45 endoscopic negative IDA patients, and reported that CE was superior to CT enteroclysis (diagnostic yield of 77.8% vs 22.2%; p < 0.001). CE was found to be better for detecting flat lesions [106]. A meta-analysis of 14 studies comparing the yield of CE with push enteroscopy for evaluation of OGIB reported higher diagnostic yield for CE (63% vs 28%, p < 0.00001) [83]. CE had a higher yield for detection of vascular and inflammatory lesions but had no benefit in detection of tumours [83].

The performance of CE has also been compared with deep enteroscopy techniques. A recently published meta-analysis reported no difference in pooled diagnostic yield of CE vs DBE (62% vs 56%, p = 0.16); however, diagnostic yield of DBE significantly increased to 75% if it was performed after a positive CE, whereas it was only 27.5% if the previously performed CE was negative.[107] One study compared CE with intra-operative enteroscopy (IOE) in 47 patients and CE identified lesions in 100% of the patients with ongoing overt bleeding whereas the diagnostic yield was 67% in patients with previous overt bleeding, and 67% in patients with obscure-occult bleeding [108]. This signifies that the diagnostic yield of CE depends upon the pattern of bleeding and is highest in patients with obscure-overt bleeding.

Evaluation of patients of OGIB with negative CE is a challenge and some studies have suggested the role of repeat CE in these patients. Jones *et al.* studied repeat CE examination in 24 patients with most common indication for repeat study being recurrent gastro-intestinal bleeding and limited visualization on the initial study. On repeat CE, additional lesions were detected in 75% of patients and this lead to change in patient’s management in 62.5% of patients [109]. In another study, out of 676 patients eighty-two underwent repeat CE examination and positive findings were detected in 55% of the patients and this lead to change in management in 39% patients. The main indications of repeat CE examination in this study were recurrent GI bleeding, iron deficiency anaemia and a previous incomplete study [110]. Viazis *et al.* studied 293 patients of OGIB, in which seventy-six patients were subjected to repeat CE examination due to non-diagnostic first test. Factors which significantly predicted the diagnosis were the change of the bleeding presentation from occult to overt, and the drop in haemoglobin of 4 g/dL or more [111]. One study reported that back to back CE within 24 hours increases the diagnostic yield in patients of OGIB with overall mean lesion-detection rates of the first and second CEs being 42.2% and 64.6%, respectively [112]. Various factors which increases the diagnostic yield of CE are patients with severe bleeding, increasing age , longer small bowel transit time, and if performed within 48 hours of bleeding [53, 113-115]. Patients with initial negative CE may have a variable rate of rebleeding from 5.6% to 45.1% in various studies and therefore these patients require close observation and regular follow up [116, 117].

**Deep enteroscopy** is being used for the complete examination of the small bowel by using double balloon enteroscopy (DBE), single balloon enteroscopy (SBE) and spiral enteroscopy (SE). All these endoscopes have both diagnostic and therapeutic potential and require an over tube for advancement of the scope. In a systemic review by Xin *et al.* which included 12,823 DBE procedures, the mid gastro-intestinal bleeding (MGIB) was the most common indication for DBE (62.5%) and the pooled detection rate was 68% (62.9-72.8%). The lesions detected were vascular (40.4%), inflammatory (29.9%), neoplastic (22.2%), diverticulum (4.9%) and others (2.7%) [118]. The pooled minor and major complications rate were 9.1% and 0.72%, respectively with major complications being perforation, acute pancreatitis, bleeding and, aspiration pneumonia. The complication rate has been described more commonly in therapeutic DBE (4.3%) as compared with diagnostic DBE (0.8%) [119].

SBE has a single balloon at tip of overtube and has similar diagnostic and therapeutic yield [120]. The complete visualization is possible in 11% of the patients, in contrast to 18% in DBE [121]. However, in another study complete examination of the small bowel was not possible in any patient by SBE [122]. SBE appears to be safe, and complication rate is low and comparable with DBE.

Spiral endoscopy has a spiral over tube of 118 cm length and at distal end there is 5.5 mm raised helix of 21 cm length. It can be fitted on any of the deep enteroscope or paediatric colonoscope. When compared with DBE, SE reduces the examination time but the depth of insertion is greater in DBE; however, another large study showed depth of insertion to be greater in SE, but had similar diagnostic and therapeutic yield [123, 124]. In a prospective, randomized, single centre trial of 26 patients, DBE perform better with regard to the depth of insertion or the rate of complete enteroscopies achieved, but required more time as compared with SE [125]. When SE was compared with SBE, SE was found to be having greater depth of insertion but similar diagnostic yield and procedural time [126]. The risk of hyperamylasemia (20%) was common after SE but no pancreatitis was reported in a cohort of 32 patients [127]. In summary, most parameters are comparable among all the three deep enteroscopy techniques; however, the procedural time is shorter in SE, as compared with SBE and DBE. The complete enteroscopy rate is higher in DBE than that of SBE and SE; but the clinical impact of complete enteroscopy needs further evaluation (Table 2).

**Table 2.** Comparison of DBE, SBE and SE

	DBE	SBE	SE
Complete enteroscopy	0–92%	15%–25%	8%
Time to completion	45–119 min	15–99 min	20–100 min
Depth of insertion-oral	240–360 cm	133–256 cm	176–250 cm
Anal	102–140 cm	73–163 cm	75–136 cm
Diagnostic yield	41–80%	47%–74%	22–75%
Therapeutic yield	42–97%	14.6–42%	13–70%
Complications			
Diagnostic	<1%	1%–11.7%	0.3%
Therapeutic	4.3%	4.8%	
Rebleeding	0–91%	39.5–55.9%	26%
Invasiveness	Yes	Yes	Yes
Sedation required	Yes	Yes	Yes

DBE: double balloon enteroscopy, SBE: single balloon enteroscopy, SE: spiral enteroscopy.

Intra-operative enteroscopy is a last resort and gold standard for evaluation of OGIB. Due to the availability of CE and deep enteroscopy, it is infrequently used in current practice. Indications for IOE are when small bowel lesions have not been localised with other techniques or cannot be treated by endoscopic or angiographic embolization or when patients condition does not allow non-invasive diagnostic evaluation [128]. IOE can be accessed either by open laparotomy or by laparoscopic assisted technique [129]. The different approach for IOE that can be used are- the transoral, transanal, through the enterotomy site or combined [128]. The endoscopes which are preferred for this purpose are the gastroscope or paediatric colonoscope and to reduce the risk of infection they should be sterile. The surgeon colleague helps by telescoping the small bowel over the scope. Because of infrequent indications and invasiveness, caution should be exercised before taking the patient for IOE [128].

In a study comparing four forms of small bowel endoscopy (IOE, CE, PE and DBE) the diagnostic yield was found to be 88%, 34.6%, 34.5% and 43% respectively [130]. This study emphasises the value of CE as first line investigation as it has a comparable diagnostic yield with respect to PE and DBE while being less invasive and more tolerable.

### Management

The small bowel bleeding can be managed by conservative, radiological, pharmacologic, endoscopic and surgical methods, depends upon the indications, expertise and availability. A patient with acute overt ongoing bleed needs resuscitation, localization of bleeding by scintigraphy, angiography or deep enteroscopy followed by therapeutic procedures. For occult or intermittent overt bleeding, localization should be done by endoscopic or radiological methods, and it should be followed by definite therapy and iron supplementation.

The various therapeutic endoscopic modalities available are argon plasma coagulation (APC), electrocoagulation, injection sclerotherapy, laser photocoagulation, haemoclip placement, and endoscopic band ligation. The endoscopic therapy of vascular lesion can be decided on the basis of Yano-Yamamoto classification, which categorizes vascular lesions in six categories [131].

Type 1a- punctulate erythema (<1 mm), with or without oozing
Type 1b- patchy erythema (a few mm), with or without oozing
Type 2a- punctulate lesions (<1 mm), with pulsatile bleeding
Type 2b- pulsatile red protrusion, without surrounding venous dilatation
Type 3- pulsatile red protrusion, with surrounding venous dilatation
Type 4- other lesions not classified into any of the above categories.

Types 1a and 1b are considered angioectasias and can be treated by cautarization. Types 2a and 2b are Dieulafoy’s lesions; managed with haemoclip placement or surgery. Type 3 represents an arteriovenous malformation; require haemoclip, banding, sclerosant or surgery.

Vascular lesions that are diffuse or are present in patients who are unfit for invasive therapies can be treated with various pharmacologic agents. Hormonal therapy (oestrogen and progesterone) has been found to beneficial in various studies; however, single randomized trial and a retrospective case control study did not show any benefit of hormonal therapy and therefore current evidence does not support the role of hormonal therapy [132-136]. Thalidomide, a VEGF inhibitor that inhibits angiogenesis has been used for recurrent, refractory or chronic gastro-intestinal blood loss due to angiodysplasia [137]. The studies have shown decrease requirement of blood transfusion and increase in haemoglobin after treatment with thalidomide [138, 139]. There is single open label randomized controlled trial, which compared the efficacy of 100 mg thalidomide (n = 28) with 400 mg iron (n = 27) daily for 4 months and followed up these patients for at least 1 year [140]. The effective response rate was defined as the decreased in bleeding episode by ≥ 50% in the first year of the follow-up period. Effective response rate was significantly higher in thalidomide group as compared with iron group (71% vs 3.7%, respectively; p < 0.001). There was also significant decrease in blood transfusion, overall hospitalization, hospitalization for bleeding episodes and level of VEGF. However, adverse events were higher in thalidomide group as compared with iron group (71.4% vs 33.3%, respectively). The common adverse effects were fatigue, constipation, dizziness, abdominal distension and, peripheral oedema. Octreotide has also shown benefit in various case series, but there is no published randomized control trial. The postulated mechanism of action is multipronged and includes improved platelet aggregation, decreased splanchnic blood flow, increased vascular resistance and inhibition of angiogenesis [141]. A meta-analysis by Brown *et al.* which included 62 patients showed octreotide decreases the need for blood transfusion and can be used in patients with refractory bleeding, inaccessible lesions and in patients at high risk for other interventions [142]. The long acting intra muscular octreotide has also been tried and found to be beneficial in treating angiodysplasias [143, 144].

Dieulafoy’s lesion can be treated with endoscopic and interventional radiological techniques, depending on the availability and expertise [31]. The various endoscopic techniques used in the treatment are argon plasma coagulation, haemoclips application, injection therapy and combination of all these [29]. Surgery is required in recurrent bleeding or failed endoscopic treatments [29, 31]. Ectopic varices within the reach of endoscopy can be treated with endoscopic haemostatic methods. Those beyond the reach of endoscopy will require embolization via intervention radiological techniques or surgery [32]. The role of vasoactive drugs in acute bleeding episodes and beta blockers for primary and secondary prophylaxis is not clear. The small intestinal ulcers can be treated with endoscopic techniques or surgery in case of recurrent bleeding. Ulcers due to specific aetiology require treatment according to the aetiology. NSAIDs should be stopped in bleeding due to NSAID-induced ulcers. Haemobilia can be managed conservatively in patients with haemobilia due percutaneous transhepatic cholangiography and liver biopsy [145]. It can also be managed by transcatheter arterial embolization (TAE), which can achieve haemostasis in 75 to 100% of cases [76]. Percutaneous thrombin injection should be considered as an option where angiography has failed or is contraindicated [146]. The indication for surgery is failed TAE or the underlying cause of haemobilia that itself is the indication for surgery [76]. The therapeutic options for haemosuccus pancreaticus are TAE and surgery. The success rate of TAE is approximately 80 to 100%. Recurrent bleeding may occur in about 17-37% of patients, which can be managed with repeat TAE or surgery [147]. The surgery is indicated when TAE fails or other indication of surgery (pseudocyst drainage) [148]. The success rate of surgery is ranging from 70 to 85% with mortality rates of 20-25% and rebleeding rates of 0-5% [149]. It is important to treat the underlying pseudocyst to prevent recurrence of bleeding [150].

**Figure 7.**
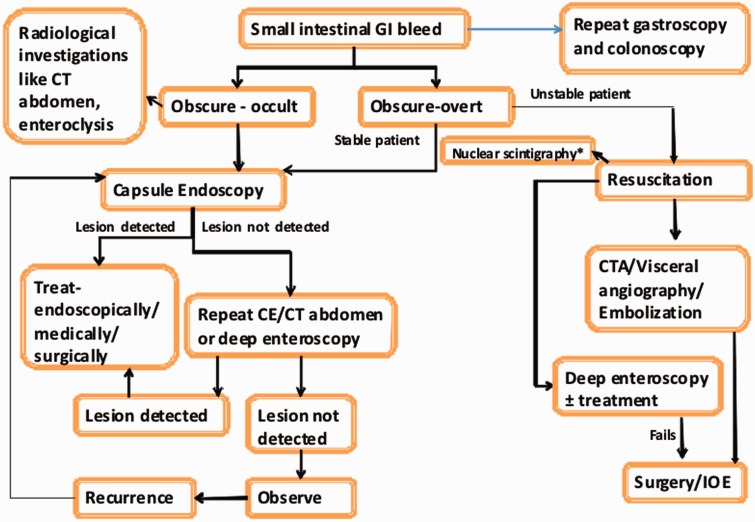
Our approach to small intestinal bleeding *Meckel’s scan in young patient; CE: capsule endoscopy, CECT: contrast enhanced CT, CTA: CT angiography, IOE: intra-operative enteroscopy.

As previously mentioned management of small bowel bleeding is tempered by the presentation and it is important to resuscitate and identify the source quickly in patients with overt bleeding. However CE is often the first modality to be utilised after a negative upper and lower endoscopy irrespective of the presentation (Figure 7) [151].

Although the role of surgery has declined with technological advances, however it has still role in some patients with small bowel bleeding. Surgery is usually required in case of life-threatening bleeding, failure of other haemostatic techniques, haemodynamic instability and clinical deterioration, recurrent bleeding, and lesions beyond the reach of endoscope. Surgical resection or excision is the treatment of choice for small bowel tumours. The recurrent diverticular bleeding and aortoentric fistula require surgical intervention.

## CONCLUSIONS

The small intestinal bleeding is rare cause of gastro-intestinal bleeding. The vascular lesions are the most common implicated lesions for the small intestinal bleeding. Capsule endoscopy and deep enteroscopy has made it easy to diagnose the causes of small intestinal bleeding. The computed tomography is more useful in detecting mural and extra-intestinal lesions. There is paradigm shift in managing the vascular lesions after advent of the double balloon enteroscopy. The pharmacological treatment can be helpful in recurrent, refractory, inaccessible angiodysplasias lesions and in patients at high risk for other interventions. The role of surgery is declining, however it is last resort in failed endoscopic treatments and recurrent bleeding.

**Conflict of interest:** none declared.
